# Chemical Camouflage– A Frog's Strategy to Co-Exist with Aggressive Ants

**DOI:** 10.1371/journal.pone.0081950

**Published:** 2013-12-11

**Authors:** Mark-Oliver Rödel, Christian Brede, Mareike Hirschfeld, Thomas Schmitt, Philippe Favreau, Reto Stöcklin, Cora Wunder, Dietrich Mebs

**Affiliations:** 1 Museum für Naturkunde Berlin, Leibniz Institute for Research on Evolution and Biodiversity, Berlin, Germany; 2 Theodor-Boveri-Institute (Biocenter of the University), Department of Animal Ecology and Tropical Biology (Zoology III), Würzburg, Germany; 3 Atheris Laboratories, Bernex, Switzerland; 4 Institute of Legal Medicine, University of Frankfurt, Frankfurt/M., Germany; 5 Medizinische Klinik und Poliklinik II, Zentrum für Experimentelle Molekulare Medizin, Würzburg, Germany; 6 Ecological Networks, Biology, Technische Universität Darmstadt, Darmstadt, Germany; Arizona State University, United States of America

## Abstract

Whereas interspecific associations receive considerable attention in evolutionary, behavioural and ecological literature, the proximate bases for these associations are usually unknown. This in particular applies to associations between vertebrates with invertebrates. The West-African savanna frog *Phrynomantis microps* lives in the underground nest of ponerine ants (*Paltothyreus tarsatus*). The ants usually react highly aggressively when disturbed by fiercely stinging, but the frog is not attacked and lives unharmed among the ants. Herein we examined the proximate mechanisms for this unusual association. Experiments with termites and mealworms covered with the skin secretion of the frog revealed that specific chemical compounds seem to prevent the ants from stinging. By HPLC-fractionation of an aqueous solution of the frogs' skin secretion, two peptides of 1,029 and 1,143 Da were isolated and found to inhibit the aggressive behaviour of the ants. By *de novo* sequencing using tandem mass spectrometry, the amino acid sequence of both peptides consisting of a chain of 9 and 11 residues, respectively, was elucidated. Both peptides were synthesized and tested, and exhibited the same inhibitory properties as the original frog secretions. These novel peptides most likely act as an appeasement allomone and may serve as models for taming insect aggression.

## Introduction

Interspecific associations ranging from mutually beneficial, neutral to parasitic are considered to be an important driver of evolution and are particularly well studied in host-parasite and plant-insect interactions [1], [2], [3]. Invertebrates of various families, orders or even classes are often living together such as ants and other insects [4]. These associations are mostly parasitic or neutral, mutualistic combinations, such as ant-aphid interactions being the exception [5]. Neutral or mutualistic associations between vertebrates and invertebrates are known from marine ecosystems, e.g. cleaner association of reef fishes and crustaceans [6], but are less known from terrestrial habitats; although the benefits of such associations are manifold and may comprise protection from predators or parasites [7], access to particular food sources [8] or a unique shelter [9].

Anuran amphibians are preyed upon by a variety of vertebrate and invertebrate species [10]. To avoid predation frogs developed a wide range of defence strategies such as concealing or aposematic colouration, special encounter behaviour, noxious or toxic skin secretions, but also seeking a safe shelter such as retreating to burrows or living underground [11]. The associations of frogs with invertebrates such as spiders (e.g. [12], [13], [14], [15], [16], [17]) or ants [18], [19], [20], which are potential frog predators [11], [21], might be a further very particular strategy to avoid predation. The proximate mechanisms facilitating these associations, however, are not yet understood.

The medium-sized (40–60 mm) microhylid frog *Phrynomantis microps* inhabits the savanna regions of West Africa [22], [23] where it hides in burrows or empty termite mounds during the day and the dry season. However, the frog was also observed to occupy and live essentially unharmed in the nest of the highly aggressive ant *Paltothyreus tarsatus* ([24]; Appendix S1 and S2). This ponerine ant species has large workers, reaching 25 mm body length and builds huge underground nests. A colony may consist of several hundred to several thousand workers [25]. They have powerful mandibles and a sting connected with a venom gland [4]. As hunters and scavengers the ants predominantly prey on larger arthropods [26], but also on frogs and other small vertebrates (MOR, *unpubl. obs*.). The benefit for the frogs to live in the underground ant-nest is evident: (1) It provides a safe retreat and protection from (other) predators, since the ants effectively defend their nest against any intruder by fiercely stinging and injecting powerful venom, and (2) the nests produce a constantly humid atmosphere enabling the frogs to survive the long dry season.

Ant nests are known to be inhabited by many other, mostly arthropod, species, which need to evolve mechanisms that enable them to circumvent the colonies' defence strategies [4]. Some species can imitate a colony member due to the chemical resemblance of their cuticular hydrocarbon profiles (e.g. [27], [28], [29], [30]). Other species exhibit only a very small amount of hydrocarbons and thus use their “invisibility” to be integrated into the host colony [31]. Furthermore, deterrents, propaganda and appeasement substances can be used by intruders to invade ant nests without being harmed [31], [32], [33], [34].

Previously, it was demonstrated that the skin secretion of *P. microps* prevents attacks of the ants [24]. Other frog species, when presented to the ants, are immediately attacked and stung. Termites (*Macrothermes bellicosus*) and anurans which are otherwise killed right after contact with the ants are not stung when they had been wetted with the skin secretion of *P. microps* [24]. In the present study we identified the chemical nature of some compounds of the frog's secretion which inhibits the aggressive behavior of the ants. These compounds belong to a hitherto unknown class of peptides.

## Materials and Methods

### Ethics Statement

No Institutional Animal Care and Use Committee (IACUC) or ethics committee approved this study as this was not required by German law. According to the German Protection of Animal Act (“Tierschutzgesetz”, latest adapted on 9 December 2010; http://www.gesetze-im-internet.de/tierschg/BJNR012770972.html; assessed on 12 June 2013) painless experiments and observations with vertebrates neither require permission nor disclosure (§ 1/§ 7 TierSchG). The vertebrates involved, *Phrynomantis microps*, experienced no pain, suffering, complaints or harm. The German Protection of Animal Act only applies to vertebrates, decapods and cephalopods. Experiments with other invertebrates, here: beetles (larvae of *Tenebrio molitor*), termites (*Macrothermes bellicosus*) and ants (*Paltothyreus tarsatus*), are not subject for approval. None of the vertebrates or invertebrates involved in our study are protected by any national or international law. All our work complied with the guidelines for the use of live amphibians and reptiles in field research compiled by the American Society of Ichthyologists and Herpetologists (ASIH), The Herpetologists' League (HL) and the Society for the Study of Amphibians and Reptiles (SSAR).

Research and collections permits in Africa were issued by the respective ministries as well as government bodies in Bénin: Faculté des Sciences Agronomiques, Département d'Aménagement et de Gestion de l'Environnement, Laboratoire d'Ecologie Appliquée, Université d'Abomey-Calavi on behalf of the Centre National de Gestion des Réserves de Faune and the Ministère de l'Environnement et de la Protection de la Nature; and Côte d'Ivoire: Ministère de l'Environnement et du Cadre de Vie, Direction de la Protection de la Nature; Ministre de l'Enseignement Supérieur et de la Recherche Scientifique, Direction de la Recherche; Ministère de la Construction et de l'Environnement, Direction de la Protection de la Nature; Ministère de l'Environnement et de la Forêt, Direction de la Protection de la Nature; Société de Développement des Forêts.

### Animals

Frogs (*Phrynomantis microps*) and ants (*Paltothyreus tarsatus*) were collected in the Pendjari Biosphere Reserve (PBR), a savanna area in northern Bénin, West Africa (N 10°30′–11°30′, W 0°50′–2°00′). Thirteen adult frogs were taken alive to the University of Würzburg (UW), Germany, where they were kept in terraria (12/12 light cycle; 25±1°C), fed with crickets, *Achaeta domestica*, and *Drosophila* sp. flies. Mealworm larvae, *Tenebrio molitor*, were purchased from the pet trade. Termites, *Macrotermes bellicosus*, were excavated either in PBR or the Lamto Faunal Reserve (LFR), a savanna in central Côte d'Ivoire, and immediately used in field experiments. The ants used in the field experiments were excavated and housed in plastic containers (31.0×41.0×23.5 cm; filled with savanna soil) at least one week prior to the tests. Ants from different colonies were kept separately, supplied with water and fed with termites and locusts. Two excavated colonies with queens (227 and 258 workers, respectively) were taken to UW, kept in special containers for ant colonies (12/12 light cycle; 25±1°C; for method see [35]) and fed with *Achaeta domestica* and *Tenebrio molitor* larvae. One of the ant colonies successfully reproduced under laboratory conditions. These ants were used for the laboratory experiments.

As we had only termites available in Africa and only mealworms in Germany we first tested if mealworms are suitable for our approach by repeating the experiments of Rödel & Braun [24]. We therefore coated mealworms (UW) or termites (PBR) with either pure water (all controls), with lyophilized skin secretions of *P. microps* (mealworms), or rubbed on *P. microps* (termites). Subsequently, they were offered to the ants and time from first contact to stinging was recorded. After confirming the results from [24] with both insect species we aimed to identifying the compounds of the frog's skin secretions responsible for the lack of aggressive ant behaviour.

### Collection and analysis of the skin secretion

Frog secretions were collected by placing *Phrynomantis microps* in a beaker (250 ml) containing 10 ml distilled water to stimulate skin secretion by shaking for two min. The frogs' secretions were frozen and lyophilized. For analysis, the sample was dissolved in 0.1% trifluoroacetic acid (TFA) and centrifuged for 10 min at 3,000 rpm. The supernatant was fractionated by reversed-phase HPLC (LiChroCART column, 125×4 mm, Merck, Darmstadt, Germany) which was eluted with a linear gradient of solvent A (0.1% (v/v) TFA in water) to 100% solvent B (60% (v/v) acetonitrile in 0.1% (v/v) TFA in water) over 60 min at a flow rate of 0.5 ml/min using the Agilent 1200 Series HPLC system. The eluant was monitored at 220 nm. The fractions with a visible absorbance were manually collected, lyophilized and subsequently applied to mealworms for testing in the laboratory.

### Peptide analysis and synthesis

For analysis by liquid chromatography linked to time-of-flight mass spectrometry (LC/TOF-MS), the positively tested samples exhibiting no or significantly delayed stinging behaviour of ants (see below), were dissolved in 100 µl acetonitrile:water (50∶50, v/v) containing 0.1% formic acid. 2 µl of the solution were analyzed using the Agilent 1100 Series HPLC system interfaced to an Agilent 1100 Series-TOF system (Waldbronn, Germany) operated in positive electrospray ionization mode (ESI). The amino acid sequence of the two active peptides was automatically analyzed by ESI-MS/MS using a Q-TOF micro mass spectrometer (Waters/Micromass, Miford, MA, USA) by nanospray in the positive ionisation mode. The native sample was dissolved in water/acetonitrile/formic acid (v/v/v 49.8/50/0.2), loaded in a PicoTip® emitter (New Objective, Woburn, USA) and analyzed by ESI-MS. Collision induced dissociation was manually adjusted for the best fragmentation pattern. The multiply-charged spectrum was transformed into a singly-charged axis using the MaxEnt3 option from Masslynx 4.0 (Waters, Milford) to allow *de novo* sequence analysis.

The peptides were synthesized using a multichannel peptide synthesizer adapted to Boc chemistry. Classical Boc protected amino acids were used during the assembly and deprotection. Leucine residues were chosen when isomeric amino acids leucine and isoleucine were identified by ESI-MS/MS. Cleavage from the resin was performed with HF. After purification by reverse-phase HPLC, the peptide purity and integrity were controlled by ESI-MS. The final product was found to be homogeneous upon standard HPLC and MS quality control analyses; their molecular weights were identical to those of the native peptides.

### Bioassays in laboratory

The entire lyophilized skin secretions of *P. microps*, 32 HPLC fractions of the lyophilized skin secretions were separately dissolved in distilled water and applied to mealworms until they were coated with the solution. Coated mealworms were placed into a plastic arena containing 30 workers of *P. tarsatus* from the laboratory colony (surface of plastic containers: 41.0×31.0 cm). Time was recorded from the first antennal contact of an ant until the mealworm was stung by one of the ants. The experiment was terminated after 5 min when no stinging occurred. Mealworms and ants involved in interactions were removed from the arena before a new trial was started and not used again. Removed ants were replaced by naïve workers to keep the number of test ants constant. The size of the ant colony (app. 250–300 individuals) limited the number of possible replicates when testing the various HPLC fractions.

### Field experiments

One mg of either of the two synthesized peptides (A & B) and a combination of both were kept in Eppendorf-tubes at 4°C. In LFR we excavated *P. tarsatus* workers and small *M. bellicosus* soldiers from colonies living in the wild. We used *M. bellicosus* termites as test organisms in the field as (1) under natural conditions they are killed by *P. tarsatus* within a few seconds after encounter, (2) were always about the same size and (3) were available in large numbers. After 2–3 days of acclimatisation 60 ants were placed in a plastic arena with a diameter of 45 cm and a central hiding place. The peptides were dissolved in 1 ml of mineral water. A termite soldier was completely immersed in one of the four solutions, i.e. mineral water (control group) or peptide solutions A, B or A+B, respectively. The termite was then placed into the arena. We stopped the time from first contact of an ant with the termite. In contrast to the above experiments we exclusively recorded the time period during which an ant and a termite were in direct contact (recording stopped when contact was interrupted). The experiment was terminated when a termite was either stung or survived 20 sec of contact. Ants and termites being in contact with each other were removed after each trial. Ants were added to the arena when their numbers were below 50, in order to keep the ant numbers almost constant. Statistical analyses of the data were performed using BiAS.8.2-2006 and R 2.14.1.

## Results

### *In vivo* experiments

**Figure 1:**
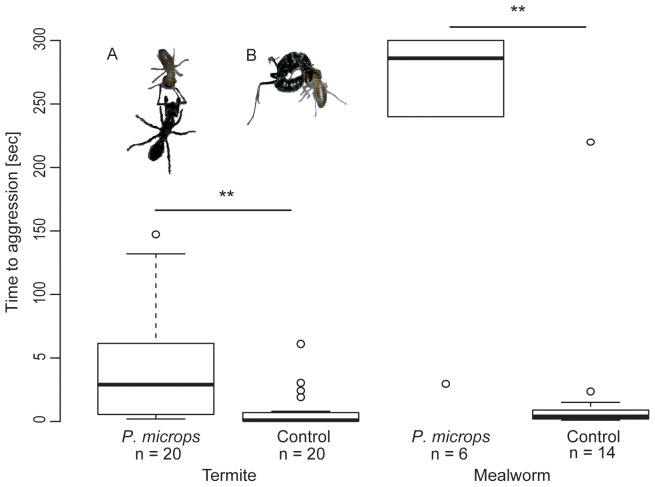
Time from first ant, *Paltothyreus tarsatus*, contact with termites (left; inlet A) or mealworms (right), coated with the skin secretion of *Phrynomantis microps*, until stinging (inlet B). Control groups are termites or mealworms coated with water. Boxplots show the median and the interquartiles of time from first ant contact with a termite or mealworm until stinging. Coated insects were stung significantly later than control insects.

The ants when encountering termites act with antennating followed by immediate biting and stinging (Fig. 1, inlets A & B). Under experimental conditions the ants stung untreated termites right after contact (median = 1.0 sec, n = 20). However, they stung significantly later when a termite was coated with the skin secretions of *P. microps* (termite rubbed over frog; median time from ant contact to stinging: termites*_P. microps_* = 29 sec; termites_control_ = 1 sec; Wilcoxon-Test: W = 335.5, p<0.001, n*_P microps_* = 20, n_control_ = 20; Fig. 1).

When the mealworms were coated with the lyophilized skin secretion of the frog, they were stung much later (median = 286 sec, n = 6) compared to control insects wetted with distilled water only (median = 4 sec, n = 14; Wilcoxon-Test: W = 83, p<.001; Fig. 1).

In both prey species previous results [24] were confirmed as the skin secretion of the frogs significantly delayed the ants' stinging behaviour. Hence both insects were used in subsequent experiments.

### Analysis of the skin secretion of *Phrynomantis microps*

The frogs' secretions did not depend on particular food items (Appendix S3). Testing the presence of hydrocarbons and alkaloids in the skin secretion by gas-chromatography/mass spectrometry (GC-MS), as found in the skin of dendrobatid frogs, or of terpenoids and steroids secreted by skin glands of toads [35] using liquid-chromatography/mass-spectrometry (LC-MS-TOF) techniques proved to be negative. By HPLC-fractionation of the secretion 32 fractions were obtained and lyophilized. After dissolving in distilled water these fractions were tested in a bioassay using mealworms presented to ants reared in the laboratory.

**Figure 2:**
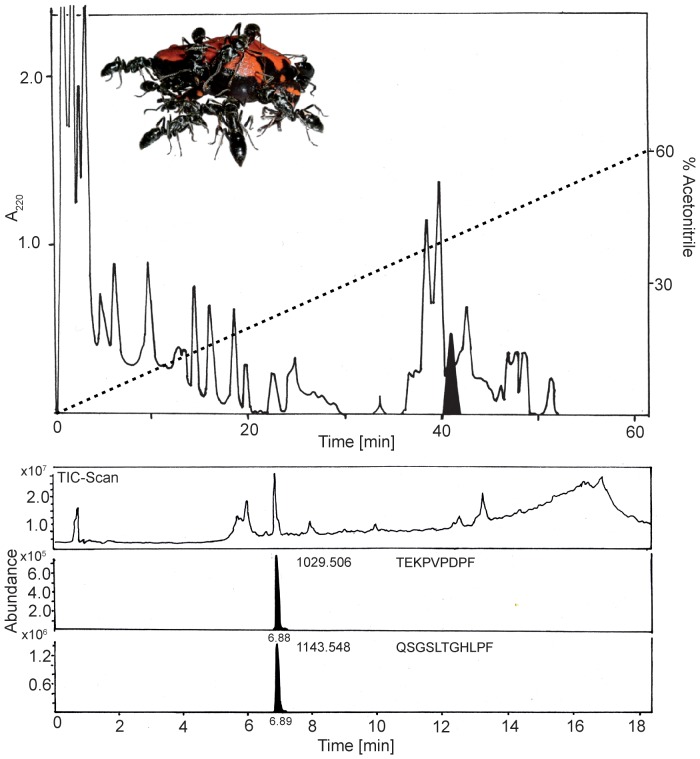
Fractionation of the skin secretion from *Phrynomantis microps* by reversed-phase HPLC on a LiChroCART column, 125×4 mm (Merck, Darmstadt) which was eluted with a linear gradient from 0 to 60% acetonitrile in 0.1% trifluoroacetic acid (dotted line) over 60 min at a flow rate of 0.5 ml/min. Absorbance was monitored at 220(above). Below: Total ion chromatogram (TIC) of liquid chromatography-time-of-flight mass spectrometry (LC/TOF MS) analysis of the HPLC fraction containing two peptides: *m/z* 1029.506 and *m/z* 1143.548 (non-protonated). *De novo* sequencing of the peptides suggested the tentative sequences as indicated in the chromatograms. Inlet picture: adult *Phrynomantis microps* examined by *Paltothyreus tarsatus* workers.

Mealworms coated with the entire lyophilized skin secretion of the frogs were stung significantly later than those coated with water only (see above, Fig. 1). Several fractions delayed the aggressive stinging reactions of the ants (results not shown); however, one fraction in particular inhibited the ants' aggressive behaviour (twice no aggressive behaviour at all; once stinging only after 206 sec). This fraction was further analysed (Fig. 2).

Liquid chromatography mass spectrometry analysis of this fraction allowed the identification of two compounds exhibiting molecular masses (*m/z*) of 1029.14 and 1143.25, respectively, suggesting a peptide structure. Due to their low amount and co-elution by liquid chromatography, their amino acid sequence was deduced by ESI-MS/MS suggesting the following, tentative primary structure: peptide A – TEKPVPDPF and peptide B – QSGSLTGHLPF. Both peptides consisting of 9 and 11 amino acids, respectively, are cysteine-free and share the common C-terminal sequence of proline-phenylalanine. No peptides exhibiting similar amino acid sequences were revealed by BLAST search of the protein database of the National Center for Biotechnology Information (NCBI, http//blast.ncbi.nlm.nih.gov) as per March 2013.

**Figure 3:**
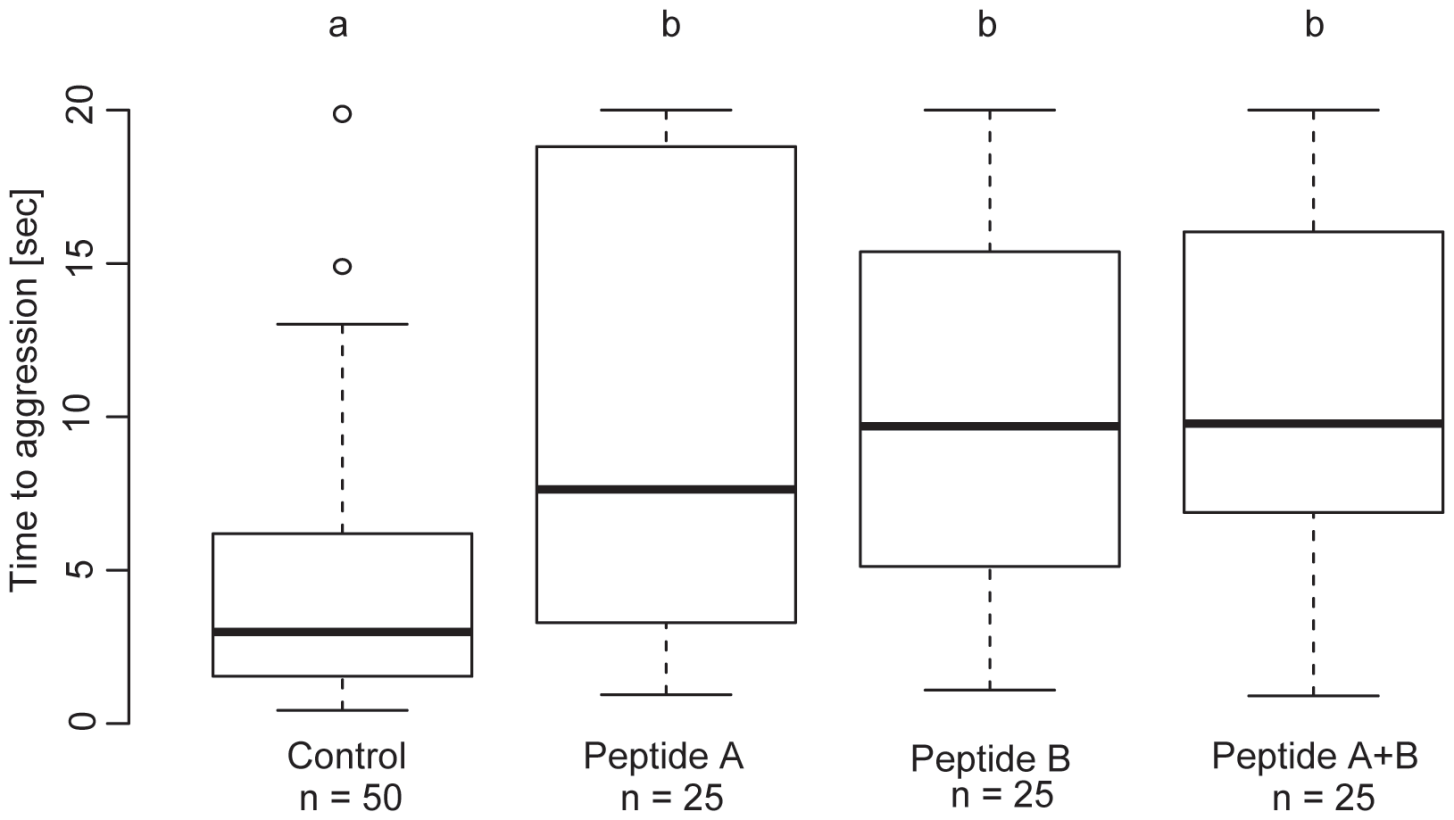
Effect of the two peptides from the skin secretion of *Phrynomantis microps* applied to termite, *Macrotermes bellicosus*, soldiers and delaying the aggressive behaviour and stinging of *Paltothyreus tarsatus* ants. Maximum observation time was 20

To confirm peptide identification, chemical synthesis of both peptides was performed by solid-phase technique and their activity was tested in the field (LFR) by presenting termites wetted with an aqueous peptide solution to *P. tarsatus*. Like the entire lyophilized skin secretion and the positive fraction from the frog skin, the synthetic peptides significantly delayed the ants' aggression (Fig. 3): Termites wetted with water only (control) were immediately stung by the ants (median = 2.9 sec; range: 0.4–20.0 sec; n = 50), whereas treatment of the termites with the peptides significantly delayed the stinging behaviour of the ants, 7.6 sec for peptide A (range: 0.9–20.0 sec), 9.7 sec for peptide B (1.1–20.0 sec), and 9.8 sec for a mixture of both peptides (range: 0.9–20.0 sec; n = 25 in each case; Kruskal-Wallis-test; H = 29.6874, df = 3, p<0.0001; DUNN-tests with ά corrected according to HOLM; treatments against control group: peptide A: Z = 3.452, p<0.01; peptide B: Z = 4.069, p<0.0001; peptides A+B: Z = 4.486, p<0.0001). The peptide treatments did not differ from each other.

## Discussion

Skin secretions of amphibians contain a huge variety of substances [37], [38] including amines, alkaloids, terpenes, steroids, peptides and proteins which are effectively used in defence against predators and microorganisms [36], [39], [40], [41]. For example more than 800 alkaloids have been detected in skin extracts of the frog families Dendrobatidae, Mantellidae and Myobatrachidae as well as of toads of the genus *Melanophryniscus* [41]. It is well accepted that these compounds are of dietary origin and derive from the frog's food sources such as ants, mites, beetles and other arthropods (e.g. [42], [43]). However, although *P. microps* feeds on ants and termites [22], our analyses did not reveal the presence of either alkaloids, terpenoids or steroids in the skin secretions.

In recent years it became evident that peptides are another important and active part of frog secretions. They have been identified in the skin secretion of various frog families [44], [45], [46], [47], [48], and show high activity against a wide array of microorganisms, including the amphibian specific fungal disease chytridiomycosis [49], [50]. However, not all investigated frog species synthesize these peptides. It has been suggested that the peptides may play a role as effector molecules in the first line of defense against infections [44], but their precise biological function is still a matter of discussion [47], [48]. These frog peptides usually comprise between 10 and 48 amino acid residues, possess a strong positive charge, contain at least 50% hydrophobic amino acids and adapt an alpha-helical conformation in solvents mimicking the environment of cell membranes [48].

The amino acid sequence of the peptides identified in the present study, which was obtained by tandem mass spectrometry, was considered as tentative. Some uncertainties such as the leucine/isoleucine differentiation could not be solved by Edman degradation due to the incomplete separation of the peptides and to their low amount in the skin secretion. However, the synthetic peptides exhibited molecular weights essentially identical to the fraction obtained from the crude skin secretion containing both peptides. In the bioassay, performed under realistic field conditions in the frog's habitat, the synthetic peptides proved to exert the same activity as the original skin secretions. The synthetic peptides are both structurally not related to antimicrobial peptides commonly present in amphibian skin secretions. Although they consist of about 50% hydrophobic amino acid residues (peptide A: 4 of 8, peptide B: 6 of 11) they are not particularly charged and represent a novel group of amphibian skin peptides. They seem to interact with the ants' antennal chemoreception in a way that the frog is either recognized as a nestmate or at least not as an intruder. When approaching the frog the ants are intensively sweeping the frog's skin with their antennae, but are not behaving aggressively, as it is always observed with other intruders. The observations that (i) *P. microps* fed with non-natural food items for prolonged periods continue producing active secretions and (ii) that even freshly metamorphosed *P. microps* are not attacked when placed in the ant colony, suggests that the presence of the peptides in the secretion is not induced, such as by contacts with ants, and not based on the uptake of particular food items, but rather an inherited property and a *de novo* synthesized product of the frog.

Although some studies have suggested that chemicals play a role in frog-invertebrate interaction [17], [19], [20], [24], this study is the first demonstrating that specific peptides play a role in this interaction in particular and in the interactions of an insect with a vertebrate in general [51]. In associations of ants with other insects such as beetles or butterfly larvae, it has been shown that certain hydrocarbon profiles in the insect cuticula are concealing the intruder and provide protection from being killed or expelled from the colony by the ants (e.g. [27], [28], [29], [30], [31]). However, to the best of our knowledge, there is no report on hydrocarbons on frog skins and skin extracts of *P. microps* using hexane as solvent with subsequent GC/MS analysis did not reveal any hydrocarbons.

Some frogs are unpalatable to predatory insects [52], including ants [53], due to their toxicity. As another *Phrynomantis* species is known to be toxic [54], [55], it could be argued that the herein observed behaviour is likewise simply a result of skin toxins. However, as social insects ants, at least in order to defend their colonies, should accept the death of some individuals. In contrast *P. microps* can freely move within *Paltothyreus tarsatus* colonies [24]. More importantly we could show that two isolated and synthesised peptides from the frogs' secretions showed the same effect on ants as observed in living frogs. This is strongly supporting the assumption that the substances are the proximate reasons of the absence of aggression in the ants when encountering *P. microps*. Most likely the peptides function as an appeasement allomone circumventing aggression of ants against the frog.

In our bioassays with termites and mealworms, a complete all-or-non-reaction was never achieved, but rather a significant reduction and/or delay in the aggressive behaviour of the ants. The lack of an all-or-non answer may be due to several reasons. Firstly, the insects can probably not be covered consistently with the peptides when wetted with a solution; in contrast to the frog where a multitude of glands provides the skin with a dense cover of secretion. Uncovered body parts of the insect, when in contact with the ants' antennae may then trigger stinging. Secondly, termite soldiers often reacted to the ants' contact with biting the latter. In such cases the information of the secretions is probably of secondary importance and as usual the ants respond with instant stinging. This is comparable to instances where *P. microps* steps on ants, which is also followed by an ant reaction of biting or even stinging [24]. In contrast mealworms, only covered in water, where also sometimes not stung. However, these beetle larvae are often very inactive, even when carried away by ants. Stinging to immobilize a potential prey is, therefore, not necessary. Lastly we cannot exclude the possibility that in addition to the two peptides identified herein, the frogs' secretion may contain other components which act solely or in combination with the peptides in preventing the ants from aggressions against the frogs. This was indicated by (i) the time differences from antennating to stinging between trials with the entire skin secretions and the peptides and (ii) several other HPLC-fractions showing similar effects in the bioassays. Unfortunately, the concentrations of the latter were far too low for further analysis. By rechromatography of these fractions their activity was mostly lost or poorly detectable. Therefore, we herein concentrated on the most active fraction in which we identified the two peptides. Nevertheless we could show that these peptides are protecting the frog from the ants' attack, particularly when they were tested under realistic field conditions in the frogs' and ants' natural habitat. The analyses and testing of potential further skin components has to be the focus of further research.

## Conclusions

The two peptides identified in this study represent a new group of compounds which not only prevent the ants to attack, but may also inhibit their feeding behaviour and most likely that of other insects. In that respect the peptides could be potentially used as models for taming insect aggression.

## Supporting Information

Appendix S1 **Video showing adult** ***Phrynomantis microps*** **moving unharmed between** ***Paltothyreus tarsatus*** **ants.**
(MP4)

Appendix S2 **Video showing** ***Phrynomantis microps*** **metamorph moving unharmed between** ***Paltothyreus tarsatus*** **ants.**
(MP4)

Appendix S3 **Indication for** ***de novo*** **synthesis of the peptides by** ***Phrynomantis microps*** *.*
(PDF)
