# Supplementary material for: Chemical Camouflage– A Frog's Strategy to Co-Exist with Aggressive Ants
Source: PLoS One. 2013 Dec 11;8(12):e81950. doi: 10.1371/journal.pone.0081950 (PMC3859521; doi:10.1371/journal.pone.0081950)
Supplement: Appendix S3 — Indication for de novo synthesis of the peptides by Phrynomantis microps . (PDF) [file pone.0081950.s003.pdf]

Rödel *et al.*:

Chemical camouflage – a frog's strategy to co-exist with aggressive ants

**Appendix S3. Secretion origin – Indication for *de novo* synthesis of the peptides by *Phrynomantis microps.***

In contrast to many other active frog substances which are acquired from prey items (e.g. [1]), e.g. millipedes and ants for alkaloids of poison dart frogs, the composition of the skin secretion of *Phrynomantis microps* is not depending on particular food items. Although being fed in captivity for one year with non-natural food items (*Drosophila* flies and *Achaeta* crickets) the skin secretions remained active when applied to mealworms. After one year captive kept frogs were transported back to the PBR and their secretions tested again (termites bathed in pure water or water where the frogs were bathed, compare [2] for exact procedure). Ant reaction to termites coated with frog secretions remained unchanged (figure S3).

In contrast dendrobatid frogs raised in indoor terraria on a diet of crickets and fruit flies do not contain detectable amounts of skin alkaloids [3]. Furthermore even freshly metamorphosed *Phrynomantis microps*, which had not yet fed on any terrestrial prey prior to the experiments, remained completely unharmed when moving within ants colonies (video S2). This supports the assumption that the frogs are able to synthesize the skin secretions by themselves.

**References**

1. Daly JW, Kaneko J, Wilham J, Garraffo HM, Spande TF, et al. (2002) Bioactive alkaloids from frog skin: Combinatorial bioprospecting reveals that pumiliotoxins have an arthropod source. PNAS 99: 13996−14001. (doi:10.1073/pnas.222551599)

2. Rödel MO, Braun U (1999) Associations between anurans and ants in a West African savanna (Anura: Microhylidae, Hyperoliidae, and Hymenoptera: Formicidae). Biotropica 31: 178−183.

3. Daly JW, Secunda SI, Garraffo HM, Spande TF, Wisnieski A, et al. (1992) Variability in alkaloid profiles in neotropical frogs (Dendrobatidae): Genetic versus environmental determinants. Toxicon 30: 887−898.

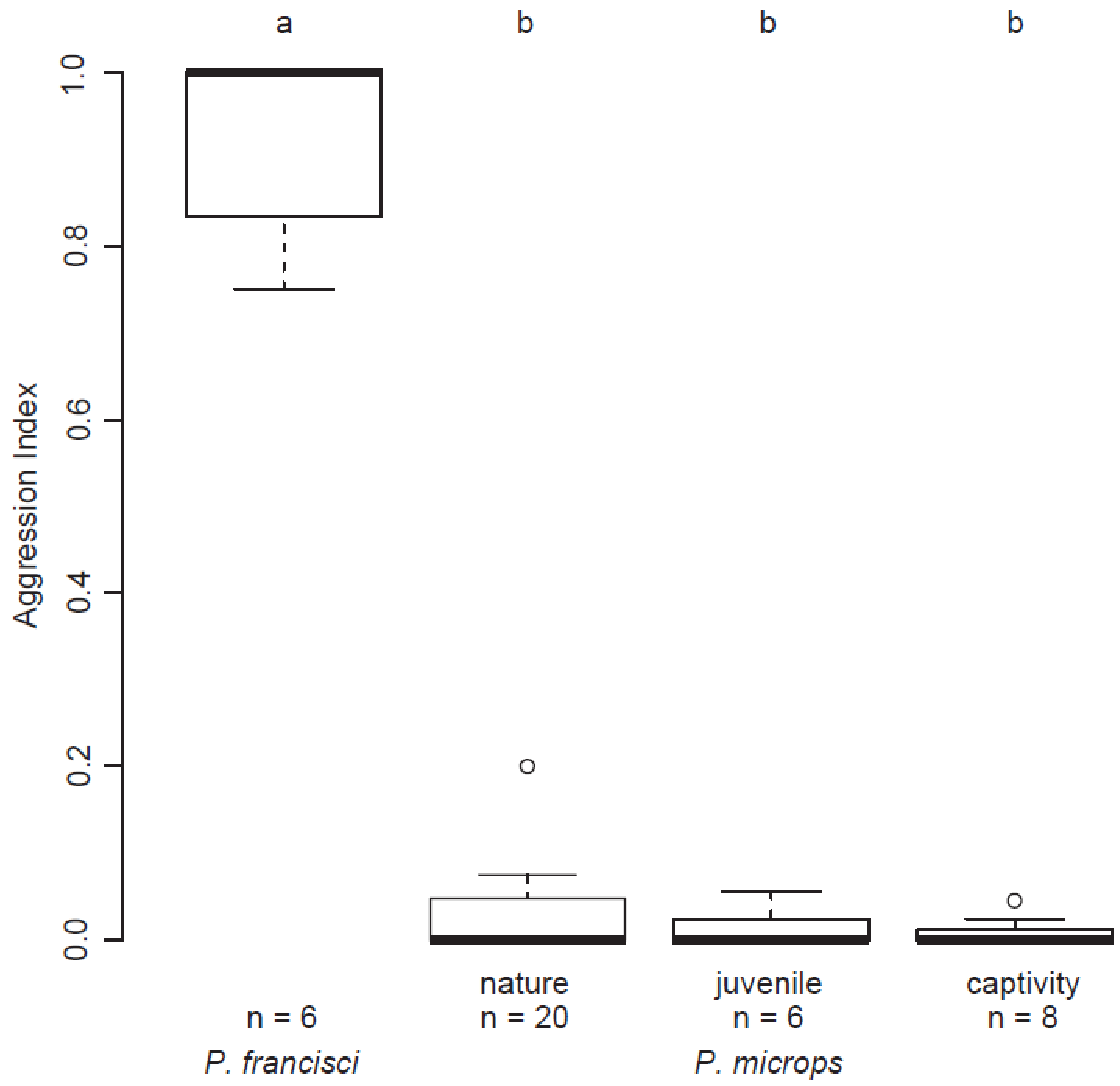


**Figure S3. Ants' behavioral responses to frogs**. *Phrynobatrachus francisci* (n = 6; Amphibia: Anura: Phrynobatrachidae), juveniles of *Phrynomantis microps* (juvenile n = adult *P. microps* kept for one year in captivity (n = 8) and adult *P. microps* from nature (n = 20). In order to display all ant responses to a frog species, we used an aggression index (*AI*). This index was compiled by defining all ant responses with "aggressive" components. Antennating as the slightest form of a contact was scored with 0, biting was scored with 0.5 and stinging as an attempt to kill was scored with 1. The *AI* was computed as following: $AI = ((N_1 * 0) + (N_2 * 0.5) + (N_3 * 1)) / N_{total}$, with $N_1$ being the sum of ants which had only antennated a frog; $N_2$ number of ants which had been bitten and $N_3$ number of ants which had been stung a frog of a particular species, age or origin. The aggression index ranges from minimum zero (no ant stung) to maximum one (every ant stung). Differences in ant aggression towards different frog species was tested with Mann-Whitney-*U*-test followed by fdr correction (different letters above bars indicate statistically significant differences between experimental groups; $p < 0.01$).
